# Ovarian dysfunction and *FMR1 *alleles in a large Italian family with POF and FRAXA disorders: case report

**DOI:** 10.1186/1471-2350-8-18

**Published:** 2007-04-11

**Authors:** Maria Giuseppina Miano, Carmela Laperuta, Pietro Chiurazzi, Michele D'Urso, Matilde Valeria Ursini

**Affiliations:** 1 Institute of Genetics and Biophysics, Adriano Buzzati Traverso, CNR, Naples, Italy; 2 Catholic University of Rome, Rome, Italy

## Abstract

**Background:**

The association between premature ovarian failure (POF) and the *FMR1 *repeat number (41> CGG_n_< 200) has been widely investigated. Current findings suggest that the risk estimation for POF can be calculated in the offspring of women with pre-mutated *FMR1 *alleles.

**Case presentation:**

We describe the coexistence in a large Italian kindred of Fragile X syndrome and familial POF in females with ovarian dysfunctions who carried normal or expanded *FMR1 *alleles. Genetic analysis of the *FMR1 *gene in over three generations of females revealed that six carried pre-mutated alleles (61–200), of which two were also affected by POF. However a young woman, who presented a severe ovarian failure with early onset, carried normal *FMR1 *alleles (<40). The coexistence within the same family of two dysfunctional ovarian conditions, one FMR1-related and one not FMR1-related, suggests that the complexity of familial POF conditions is larger than expected.

**Conclusion:**

Our case study represents a helpful observation and will provide familial cases with heterogeneous etiology that could be further studied when candidate genes in addition to the *FMR1 *premutation will be available.

## Background

Premature ovarian failure [POF (MIM 311360)] is an early ovarian dysfunction characterized by cessation of menstruation before the age of 40 years [1]. About one in every 1,000 women between the ages of 15–29 and one in every 100 women between the ages of 30–39 are affected by POF [2]. The aetiology of this disorder is complex and the underlying genetic defects are largely unknown. Studies of pedigrees with a familial history of POF suggest that this condition may be inherited as an autosomal dominant sex-limited transmission or X-linked with incomplete penetrance [3-5]. It has been estimated that ~21% of familial POF cases are associated with expanded alleles of the *Fragile X mental retardation *[*FMR1*, FXS (MIM 309550)] gene [6-8].

FMR1 is located at Xq27.3 and represents a dynamic mutational site because an unstable expansion of a trinucleotide (CGG)_n _repeat occurs at the 5' UTR of the gene [9]. Based on the number of repeats, four types of *FMR1 *alleles have been identified: 1) normal, ranging from 6 to 40; 2) grey-zone, from 41 to 60; 3) pre-mutated, from 61 to 200; and full mutation, exceeding 200. Once the *FMR1 *allele is fully mutated, hypermethylation of a nearby CpG island is triggered leading to transcriptional silencing of *FMR1 *[10]. This condition causes Fragile X mental retardation, a syndrome with macro-orchidism and behavioural abnormalities that is the most common form of inherited mental handicap in males.

Intermediate and pre-mutated *FMR1 *alleles may become unstable generating a full mutation with further expansion in the following generations, when passed from a female to her offspring [11]. Such females, also called Fragile X intermediate or pre-mutation carriers, are phenotypically normal although with an increased risk of POF [8,12]. Firm evidences exist for a significant association between *FMR1 *allele size and the age of menopause among POF females [7,8]. These studies allow improved risk assessments for the genetic counselling of familial POF manifestations where women present pre-mutated *FMR1 *alleles [12].

The aim of this study is to present a large pedigree in which two different POF disorders coexist, one *FMR1*-related and one not *FMR1*-related.

## Case presentation

### Case history

**Figure 1:**
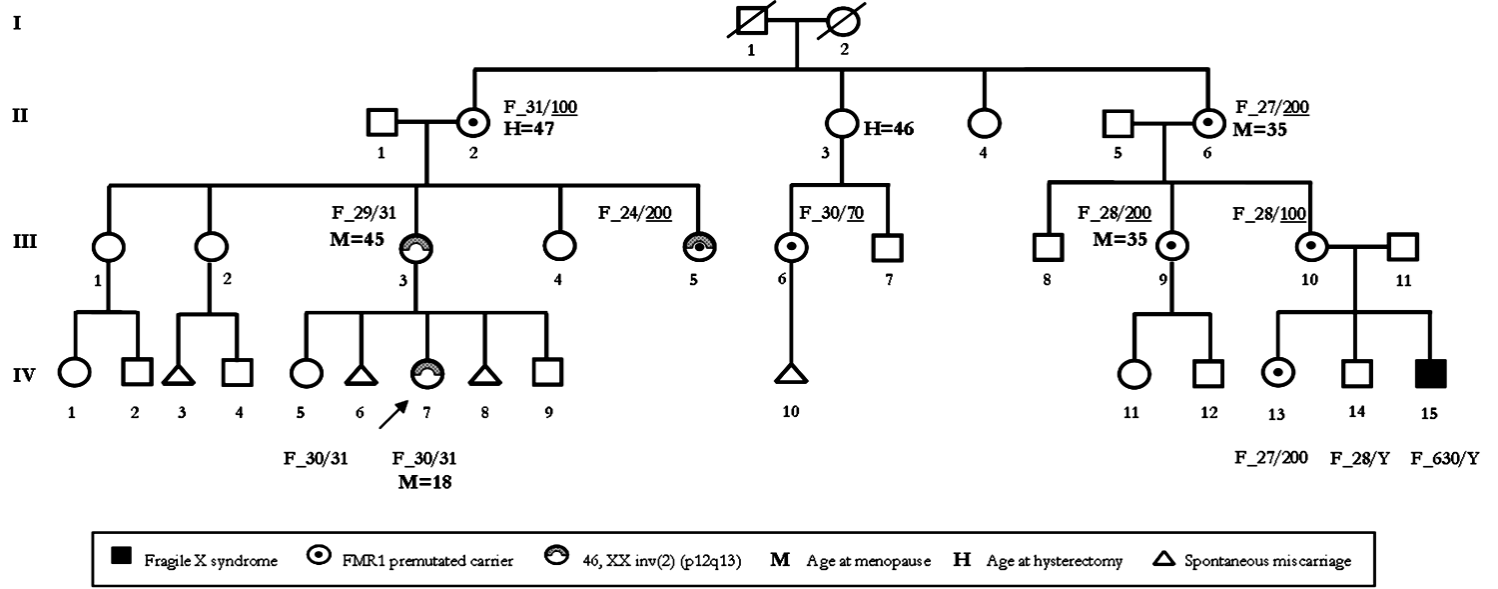
Genealogical tree of Family #_P1 presenting POF manifestation, FRAXA disorder and *FMR1 *premutated and mutated alleles. With F the genotypes of *FMR1 *alleles obtained by PCR and Southern blot assays are shown.

A large Italian three-generation kindred was brought to our attention because its members were affected by POF manifestations and FRAXA syndrome (Figure 1). All willing family members provided written informed consent under protocols approved by Declaration of Helsinki.

They were examined and classified as part of an ongoing genetic study of ovarian X-linked dysfunction. A systematic interview was conducted with thirteen family members.

According to common protocol, POF status was defined as hypergonadotrophic amenorrhoea with FSH measurements ≥ 20 IU, LH > 15 IU and cessation of menses for a duration of ≥ 6 months, at age ≤ 40 years.

The proband girl (IV:7), a 24-year-old girl, was referred because of ovarian failure with hypergonadotropic amenorrhea consistent with a diagnosis of POF (FSH measurement = 102,00 mIU/ml, LH measurement = 18,00 mIU/ml). She had a menarche at 13 years of age and had always irregular menstrual cycles until the age of 18 years when she entered menopause. Her condition was considered idiopathic because she did not show any of the known POF-related conditions such as ovarian surgery, previous chemotherapy or radiotherapy, or autoimmune disease. Pelvic ultrasonography and laparoscopic ovarian biopsies were performed. Both examinations revealed the complete absence of follicles consistent with a diagnosis of POF type B [13]. Cytogenetic analysis demonstrated that she was a carrier of an inversion of chromosome 2, with kariotype 46, XX inv(2)(p12q13) (data not shown). To our knowledge, this is a constitutional pericentric variant of chromosome 2, which originated through inversion between two chromosomal breakpoints, conserved in primates [14]. Furthermore, 2p12 and 2q13 are well known as fragile sites (FRA2L, FRA2A, FRA2B; [15]) reflecting the break-prone nature of the pericentromeric region of chromosome 2.

The female III:3 (age 49), mother of the case study, who entered menopause at 45 years, had no symptoms of POF disorder. She had a karyotype with 46, XX inv(2)(p12q13), the same inversion observed in the proband IV:7. The female IV:5 (age 25), the sister of the proband IV:7, had regular menses and a normal karyotype. Their maternal grandmother (II:2, age 75) had five daughters and underwent a hysterectomy at age 47 because she was affected by diffuse fibromatosis of the uterus. The female II:3 (age 65) had two sons (III:6 and III:7) and similarly to her sister (II:2) underwent at hysterectomy at age 46. Both II:6 (age 60) and III:9 (age 47), that are respectively the great-aunt and the aunt of the proband, suffered from a POF syndrome with amenorrhoea at age 35. The female III:10 (age 46), daughter and sister of the two POF females (II:6 and III:9, respectively) had no symptoms of premature menopause. She had two children, a 11-year-old daughter, IV:13, that was in pre-pubertal age and a young-boy of 6 years old, IV:15, with Fragile X syndrome showing severe mental retardation, behavioural problems and macroorchidism.

As referred to us, the remaining at risk females III:4 (age 38), III:5 (age 35), III:6 (age 36) had regular menses and did not suffer from POF symptoms. In one of them, III:5, the inversion of chromosome 2, inv(2)(p12q13), already present in III:3 and IV:7, was found. Regarding the females III:1 (age 57) and III:2 (age 50), both had an apparently regular fertility life and entered menopause at age 51 and 50, respectively. Spontaneous miscarriages were reported for several women (III:2, III:3, III:6) as shown in Figure 1. Unfortunately, a definite cause has been difficult to determine in each of these cases even if an association between infertility and spontaneous abortion has been proposed [16].

### Case analysis

#### FMR1 genotype repeat size

Genomic DNA was isolated from 10 ml of the peripheral blood samples in an EDTA tube, using standard procedures [17]. The *FMR1 *repeat length was determined in the family under study by fluorescent polymerase chain reaction (PCR) amplification and electrophoresis analysis on an automatic sequencer (ABI PRISM 3100, Applied Biosystem, Foster City, CA, USA). "C" and "F" primers used for the *FMR1 *gene amplification were described in Fu *et al*. (1991) [18]. The results were elaborated with Genescan 3.1 software. To validate the mutated alleles previously detected by PCR, genomic DNA was digested with EcoRI and EagI, blotted onto Hybond N+ (Amersham, Milan, Italy) and thus hybridised to the probe StB 12.3, as previously described [19].

#### X-chromosome inactivation, XCI

The XCI ratio was determined by methylation-sensitive restriction enzyme digestion of genomic DNA followed by PCR amplification and product quantification on an automatic sequencer. Digested and undigested samples were then amplified at the *AR *locus. The protocol was carried out as previously described [5].

## Results and discussion

In a large Italian kindred with a family history of POF and FRAXA disorders two different dysfunctional ovarian conditions are present, one *FMR1*-related and one not *FMR1*-related (Figure 1). The proband IV:7, is a young woman with severe POF disease as described above. Because of the presence in the family of a young-boy (IV:15) with Martin-Bell syndrome (MIM300624), we started our analysis establishing the segregation of *FMR1 *expanded alleles in the family. As expected, IV:15 had a FRAXA syndrome with an *FMR1 *full mutation with 630 repeats of the tri-nucleotide CGG. In consequences of this, an *FMR1 *study was carried out on the females related to him, either with POF manifestations (II:6, III:9, IV:7) or at risk but with regular ovarian functions (II:2, III:3; III:5, III:6, III:10, IV:5, IV:13).

Both mother and sister of the FRAXA male, III:10 and IV:13, carried pre-mutated *FMR1 *alleles. III:10 was 28/100 CGG repeat allele and IV:13 was 27/200 CGG repeat allele; while a normal brother (IV:14) carried a hemizygote 28 CGG repeat allele. His maternal grandmother (II:6) and maternal aunt (III:9), both affected with POF, were heterozygotes for premutated alleles with 27/200 and 28/200 repeat alleles, respectively.

In the mother-daughter transmission (II:6-III:10), the presence in III:10 of the FMR1 allele with 100 CGG repeat could be the result of a backward mutation or contraction upon transmission from the mother with 200 CGG allele. This phenomenon is the product of the high instability of the FMR1 CGG repeats that was found to increase with increasing of the repeat length and that, generally, occurs with higher frequency in the paternal transmission than in maternal transmission [12,20,21].

The female IV:7 (the proband) had a POF diagnosis and she was the second cousin of the FRAXA male. Therefore, we extended the *FMR1 *analysis to her familial unit. In spite of the association between POF disease and *FMR1 *expanded alleles that was ascertained in the family, the genotype of the POF IV:7 was not in agreement with this status. Indeed, she carried two normal *FMR1 *alleles, one of 30 repeats and one of 31 repeats. Her mother (III:3) and sister (IV:5), both with no symptoms of ovarian dysfunction, were heterozygotes with normal *FMR1 *alleles, 29/31 and 30/31, respectively. In contrast, her maternal grandmother (II:2) who did not have ascertained ovarian failure, carried a premutated allele with the genotype 31/100. Similarly, the aunt of the proband (IV:7), one of the daughters of II:2 who reported no history of POF, was heterozygote for premutated CGG repeats with 24/200 alleles. Another type of analysis that can give further information on the involvement of the premutated *FMR1 *gene in POF manifestation may be represented by X-inactivation studies because *FMR1*-related POF severity may be dictated by X-inactivation pattern [22]. Evaluation of X chromosome inactivation (XCI) status in the peripheral blood of the POF and risk females with normal and/or expanded *FMR1 *alleles revealed that the XCI pattern is random (data not shown). Moreover, X-inactivation was measured in cells of the blood where its status may be different in the ovarian tissue.

Based on these evidences, we assumed that in this family different factors concur to cause ovarian dysfunction: one factor is the instability of FRAXA alleles, an ascertained risk factor for the females with POF, II:6 and III:9; while an other unknown factor could operate in the young female IV:7 causing severe ovarian failure. We are unable to pinpoint exactly what the relationship is between these different conditions and how they may influence normal ovarian function.

In our opinion and accordingly to several reports [1-6,21], POF disorders should be considered as multifactorial diseases and in view of this, studying the phenotypical and genetic features of new cases could contribute to the shedding of new light on the great complexity of POF.

In conclusion, our case report represents an original observation on the coexistence within one family of different factors involved in two different ovarian failure conditions, one-*FMR1 *related and one not-*FMR1 *related. On the other hand, it can be considered an important warning for the genetic counselling of familial POF. In particular, we believe that the association of POF with expanded *FMR1 *alleles must be verified through the generations allowing the clinical counselling of familial POF and eventually the implementation of strategies to advance conception.

## Competing interests

The author(s) declare that they have no competing interests.

## Authors' contributions

MGM designed the study, collected data and drafted the manuscript. CL performed molecular and genetic studies. PC made contributions to the genetic counselling of the patients. MD interpreted experiments and revised the manuscript. MVU participated in the study design, collected data and revised the manuscript. All authors read and approved the manuscript.

## Pre-publication history

The pre-publication history for this paper can be accessed here:


